# The Emergence of Primary Anoetic Consciousness in Episodic Memory

**DOI:** 10.3389/fnbeh.2013.00210

**Published:** 2014-01-03

**Authors:** Marie Vandekerckhove, Luis Carlo Bulnes, Jaak Panksepp

**Affiliations:** ^1^Department of Experimental and Applied Psychology, Research Group of Biological Psychology, “Vrije Universiteit Brussel,”Brussels, Belgium; ^2^Department of Integrative Physiology and Neuroscience, College of Veterinary Medicine, Washington State University, Pullman, WA, USA

**Keywords:** episodic memory, anoetic consciousness, noetic consciousness, autonoetic consciousness, self, identity

## Abstract

Based on an interdisciplinary perspective, we discuss how primary-process, anoetic forms of consciousness emerge into higher forms of awareness such as knowledge-based episodic knowing and self-aware forms of higher-order consciousness like autonoetic awareness. Anoetic consciousness is defined as the rudimentary state of affective, homeostatic, and sensory-perceptual mental experiences. It can be considered as the autonomic flow of primary-process phenomenal experiences that reflects a fundamental form of first-person “self-experience,” a vastly underestimated primary form of phenomenal consciousness. We argue that this anoetic form of evolutionarily refined consciousness constitutes a critical antecedent that is foundational for all forms of knowledge acquisition via learning and memory, giving rise to a knowledge-based, or noetic, consciousness as well as higher forms of “awareness” or “knowing consciousness” that permits “time-travel” in the brain-mind. We summarize the conceptual advantages of such a multi-tiered neuroevolutionary approach to psychological issues, namely from genetically controlled primary (affective) and secondary (learning and memory), to higher tertiary (developmentally emergent) brain-mind processes, along with suggestions about how affective experiences become more cognitive and object-oriented, allowing the developmental creation of more subtle higher mental processes such as episodic memory which allows the possibility of autonoetic consciousness, namely looking forward and backward at one’s life and its possibilities within the “mind’s eye.”

## From Unknowing to Knowing Levels of Consciousness

Our evolutionary theoretical approach of understanding consciousness focuses on how lower levels of primary-process affective experience or “anoetic” consciousness emerge gradually by learning and memory into autonoetic consciousness through a progressive developmental maturation of the brain and mind (Panksepp, 1998a, 2005, 2007, 2008; Tulving, 2004, 2005; Vandekerckhove, 2009; Vandekerckhove and Panksepp, 2009, 2011). Anoetic consciousness reflects a primal state of autonomic-phenomenal awakeness, with direct experiences of oneself and the world – namely, an unknowing or “anoetic” (without explicit knowledge) consciousness consisting of perceptual, motoric-procedural and various primal emotional, homeostatic, and sensory affective states (Panksepp, 1998a; Merker, 2007). These brain-body states are all heavily dependent on subcortical neural networks, thalamic sensory relay nuclei, basal ganglia, and especially midline mesencephalic and diencephalic attentional and affective systems, which permit other neural circuits to evolve developmentally toward knowing about the world. “Knowing about the world” consists of “noetic” consciousness, based on semantic memory, relying on brain intermediate regions such as basal ganglia (amygdala, nucleus accumbens, etc.), the dorsal medial prefrontal cortex, temporal lobes, and “autonoetic consciousness,” which is supported by high-order neocortical areas (especially parietal, prefrontal, and temporal) and is mediated by self-reflective mental states within time and environmental contexts. These “knowing states of consciousness” allow for higher mental activities, especially experiential “time-travel” consisting of the re-experience of past events in the service of future plans (Vandekerckhove and Panksepp, 2011). For instance, prefrontal and temporal activations are involved in the elaboration of higher ideographic cognitive semantic and episodic representations relying on anoetic consciousness related to diverse higher-order affective self-experiences supported by limbic structures as the amygdala, insula, and anterior cingulate cortex, along with increasingly complex implicit procedural representations that involve the motor cortex.

Each higher state of consciousness depends ultimately, not only on individual lived experiences, but on those extensive primary-process, evolutionarily provided networks of universal bodily existence, through various synchronizing and desynchronizing patterns of firings of diverse neural networks that are gradually beginning to be understood. In other words, that which came first in brain-mind evolution sustains a foundational influence on all higher forms of cognitive consciousness that we cherish. This integrative multilevel view helps us understand how an enormous number of conscious experiences emerge from the continuous multi-tiered flows of affective dynamics that we experience in our daily life. Anoetic affective consciousness emerges from inherited functions of the brain, where various positive affective feelings reflect survival trajectories, while various negative affective feelings anticipate potential pathways of destruction (Panksepp and Biven, 2012).

Through the unconscious mechanisms of learning and memory, these affects are connected to environmental events in order to promote more sophisticated anticipatory behaviors through learning, and with gradual culture-promoted integration, into higher cognitive-processes that allow for internal evaluative reflections related to recall of past memories and “mind-travel” or “autonoesis.” These layered levels of consciousness are hierarchically structured whereby the higher levels of consciousness are more recently emergent states of the brain, which certainly are phylogenetically more recent and develop later in infancy (Vandekerckhove, 2009; Vandekerckhove and Panksepp, 2009).

Crucial here, in the transition from anoetic to autonoetic consciousness are the variety of fine-grained neuronal associative processes of the basal ganglia and limbic system, integrating an implicit level of unconscious neural processing that allows for learning and memory. In other words, we do not experience the brain mechanisms of learning and memory, only their results. This underlying form of primary-process form of anoetic affective consciousness which is instantiated in diverse forms of qualia, including primal affective feelings, has often been neglected in consciousness studies (Panksepp, 1998a, 2007; Merker, 2007; Vandekerckhove, 2009; Solms and Panksepp, 2012). Such primal foundational processes have often been deemed to be deeply unconscious or “unexperienced,” but this is not the case for the unconditional positive and negative feelings that arise from this level of neural processing (Panksepp, 1998a, 2005, 2007, 2008). Anoetic consciousness is constituted from “here and now” pre-reflective phenomenal experiences, not requiring higher-order forms of self-reflective consciousness subsumed by the concept of “awareness” (Solms and Panksepp, 2012). At the empirical level, evidence for this is provided by the fact that wherever in subcortical regions of the brain we can activate coherent emotional behaviors with electrical deep brain stimulation (DBS), those induced internal states are routinely “rewarding” and “punishing” in animal models (Panksepp, 1982, 1998a, 2005; Panksepp and Biven, 2012), corresponding perhaps to distinct human emotional feelings, since humans commonly report categorically distinct affective shifts when DBS is applied to such brain systems (see Panksepp, 1982). Our goal here is to provide a conceptual narrative that captures the flow of such primal brain processes that are comparatively hard to analyze explicitly with routine neuroscientific procedures in humans.

## Transitions from “Unknowing” to “Knowing” Forms of Consciousness

From our perspective, a critical distinction becomes evident: from a rudimentary state of autonomic awakeness or “unknowing consciousness” reflecting primal biologically adaptive functions, brains states first become experienced at anoetic (unknowing) levels (e.g., bodily felt affective states), from which capacities for higher forms of consciousness gradually emerged with brain-mind encephalization. First, the primal experiential levels, provided “reinforcement” mechanisms for accruing knowledge about the world (noetic states, reflecting facts about the world). Further brain encephalization, led to awareness, a higher-order “knowing” form of consciousness based on semantic and episodic memory systems, which is encapsulated by Endel Tulving’s concept of “autonoetic” consciousness, that provides access to higher meaning-making processes, reflected best in the arts, literature, and other cultural processes, that remain poorly understood at the neural level (Velmans, 1999, 2009; Block, 2005, 2007).^1^

### Implicit anoetic consciousness

The Jamesian (James, 1890) fringe of consciousness – the stream of consciousness that is so hard to describe and study has been a mainstay of affective neuroscience (James, 1890; Panksepp, 1982, 1998a, 2005, 2008) a “missing piece” that can be integrated with our emerging understanding of higher forms of consciousness (Tulving, 2004, 2005; Vandekerckhove, 2009; Vandekerckhove and Panksepp, 2009, 2011). The “anoetic” nature of implicit primal anoetic consciousness consists of a continuous underlying stream of affective and multisensorial and procedural representations in an embodied or bodily format such as an autonomic sensation that are experienced at an unreflective level. It guides associative learning processes which then gets associated with “object-relations” in the world, and thereby become ever explicit (Goldman and Vignemont, 2009; Gallese and Sinigaglia, 2011). Such learning processes yield explicit representations over which one does not exert direct volitional control until enough higher-order brain matter (i.e., neocortex) evolved to allow the rudiments of autonoetic consciousness which in humans seems to be largely verbally mediated, but which we believe also be represented as visual and perhaps other images in pre-linguistic organisms. Thus, on an implicit level, anoetic consciousness is continuously influencing mood and behavior, thereby permitting affective and perceptual experiences to gradually have noetic properties. In other words, as a continuous state of experiencing of brain and bodily affective states as associated with external perceptual information processing, anoetic affects come to be associated with world events.

The transformation of noetic experience into awareness and autonoetic possibilities, needs further amplification of here-and-now anoetic and noetic evaluative states of mind by recursive brain-mind neural systems especially by fronto-parietal networks (Dehaene et al., 2006; Dehaene and Changeux, 2011). Only if the experience gets intense enough and therefore salient, one is able to describe, and/or reflect on it (Vandekerckhove and Panksepp, 2011). The underlying autonomic and organic state processes, arising from viscerally enriched subcortical circuits extend from the mesencephalic central gray regions, through medial diencephalic regions, toward cingulate and orbitomedial frontal cortices (Panksepp, 2007) are continuously contributing to the flow and fluctuations of anoetic consciousness that provides a critical foundation and background for higher cognitive activities.

Anoetic and noetic forms of consciousness bridge the genetically provided potentials for brain-mind states with explicit experiential contents to engender higher forms of consciousness that are rich in explicit conscious awareness (i.e., knowing what one is experiencing). This progression reflects how primal phenomenal experiences can be integrated through unconscious learning and memory processes toward an ideational enrichment of higher mental life. Thereby, a variety of changes in experiences, thoughts, and actions might be attributable to past events, including priming effects, unconscious saving effects in relearning, as well as certain forms of proactive and retroactive interferences (Kihlstrom et al., 2007). Within this continuum and from the perspective of noetic consciousness, anoetic neural substrates of consciousness may come to contain many implicit stored experiences, such as early childhood experiences. They no longer come into explicit awareness at later stages of development but which, because of various affective priming processes, may still arouse affective qualia that influence higher awareness processes. For instance, anoetic experiential feelings may be triggered by perceptually related associated situations or memories and may influence behavior in daily life (Squire and Zola-Morgan, 1991), while not being recognized by the individuals themselves.

Even if is not well recognized how behaviors are accompanied by and caused by apparently free-floating affective anoetic states, something that may be inherent to such implicitly triggered anoetic states is the possibility that what may be cognitively unconscious need not be affectively unexperienced or visually unseen at a primary-process level. Indeed, many priming paradigms have shown that cueing information can still be implicitly perceived even when there is no awareness of perceiving it, and this information can be causally efficacious in enhancing target identification when attention is oriented to the location of the correct cueing stimuli (Merickle et al., 2001). Similarly, unconscious affective information such as emotional words presented at the visually undetectable levels, can reliably modify mood states (Shevrin et al., 2012). Likewise, work with neonatally decorticated animals, along with work with congenitally anencephalic children (Merker, 2007), demonstrate that one can have primary affective, sensory, and perceptual here-and-now implicit experiences with very minimal capacity to project and transform them into higher mental processes or awareness (Panksepp, 1998a, 2005; Shewmon et al., 1999). A hemi-neglect patient who suffered a lesion in the right parietal cortex will find it difficult or impossible to shift attention to the left side or to be explicitly sensitive to anything on the left side (Driver and Vuilleumier, 2001). In such hemi-neglect, there is preservation of initial sensory pathways, in line with many other studies which have shown that considerable residual processing can still take place for neglected or extinguished stimuli without reaching the patient’s awareness. Like many forms of implicit processing, this residual processing can modulate what enters into patient’s awareness. These examples also illustrate that the higher cognitive regions of the brain are not essential for the generation of anoetic consciousness. However, with the maturation of the neocortex to sustain higher mental processes, those superordinate abilities probably have an intrinsic capacity to modulate lower brain-mind processes, which may be the source of the Freudian dynamic unconscious (among the more cephalized animals), which can only be scientifically well studied in humans.

### Anoetic consciousness and the origins of “embodied aware states”

Being a “primal” embodied mental state, anoetic consciousness involves the capacity to experience body and world with sensory-perceptual immediacy and affective qualities that may emerge into higher-order awareness. It is the general representational contents of the anoetic and noetic states that determine the phenomenal character of autonoetic awareness (Churchland, 2005). The flow of these anoetic experiences does not dominate in awareness. It has no explicit object as most full-blown emotional states have (e.g., I am angry because…). In contrast, awareness involves focused attention to something specific inside or outside the organism in the world. Conscious awareness – the experience that one is experiencing – adds a new reflective dimension to consciousness (Dehaene and Changeux, 2011). Aware experience reflects the experience of something both perceptual and experientially contextualized along with an internal higher recognition of ownership of the experience. Processes of integration and differentiation play a crucial role in the process of emergence of conscious awareness achieved in short time periods, even of hundreds of millisecond (Edelman, 1993, 2003; Tononi and Edelman, 1998; Crick and Koch, 2003). These representations are the result of an underlying anoetic processing that lead to the construction of specific knowledge, an affectively motivated reaction or a thought. Anoetic consciousness becomes apparent in explicit awareness in a propositional format only when intense enough to mandate deliberative actions. Awareness is facilitated when attention is directed toward those external sensations and internal experiences that would otherwise not be perceived. In daily life, the experience of anoetic consciousness in normal subjects is less intense and more in the background of these subtle experiences, for instance feelings of familiarity in social situations. This may reflect inhibition/regulation that higher cortical ventromedial processes can impose on subcortical processes. Even if one does not talk about it, anoetic consciousness still may be expressed in body posture, gestures, and tone of speech. In the meanwhile, it is characterized by fluctuations of affective-somatosensorial background intensity as anoetic qualia are generated (Frijda et al., 1991). Such background psycho-physiological process entails continuous primary evaluations that gradually influence the affective ways in which one relates to the world, which in itself is quite distinct from various cognitive evaluations. These affective feelings (e.g., incipient desires and fears) and homeostatic ones (e.g., hunger and thirst) exist in order to help shape preferences intrinsically so as to anticipate the potential positive and negative consequences of actions. The individual may choose to do various things as underlying causes of the anoetic affective flow and associated physiological changes fluctuate (e.g., increased blood pressure, skin conductance, and heart rate). These affectively rich emotional “action tendencies” that organisms experience, facilitate not only urges to act – for instance facilitation of enthusiasm via increased brain dopamine release, but also serve as a prelude to intended actions within higher levels of consciousness (Panksepp, 2007). Associated affective arousals are endogenously further regulated by other biogenic amine transmitters such as norepinephrine and serotonin, contributing to the intensity of anoetic consciousness, that may gradually emerge into explicit awareness when raw experience is transformed into noetic consciousness that fuels autonoetic awareness. Parallel and sequential implicit internal affective and cognitive processing and re-processing enables us here, through learning and memory, to give implicit and explicit meaning to our internal experience and affective expressions within both environmental as well as social interactions in ways that can help promote cognitive thought and planning of actions.

### Underlying multimodal feedback

The flow of anoetic consciousness is dependent on sensory-perceptual signal integration and ongoing dynamic activity in core limbic and brain-stem value systems (Edelman, 1993, 2003). If subcortical signaling is altered it follows that the global interoceptive feeling changes and the tendency to adaptively process affective changes related to the world changes too. For instance, research on emotion imitation (Hennenlotter et al., 2009) showed that denervation of muscles necessary to the facial expression of emotion leads to changes in central circuitry of emotion. Deafferentation of frown area with botulinum-toxin diminishes left amygdalar activity and functional connectivity between amygdala and several brain-stem regions implicated in the control of automatic arousal (Hennenlotter et al., 2009). The impaired feedback and integration of negative affect-related signals results in diminished activity of affective networks that control the anoetic quality of one’s background experiences. Also when related brain areas are impaired, this updating function becomes impaired which normally helps thus to maintain and continuously update affective experience and the capacity to detect and interpret higher-order affective information such as own internal state learning and memory that is largely unconscious. Integral information processing in anoetic consciousness is thus continuously instantiated by multimodal feedback and large-scale neurodynamics resulting in accompanying brain neurochemical/electrical changes that regulate information processing, through presently poorly understood “laws of affect,” perhaps by conversion of silent glutamateric synapses to active ones (Panksepp and Biven, 2012).

## Anoetic Self-Consciousness Versus Self-Concepts

By bringing phenomenal experiences into reflective consciousness – we affectively experience how we “are” which may govern how we intuitively feel how we should behave in the world. These increasingly higher-order experiences are directly related to the affective conditions that inundate the core-self, a virtual neural representation of the body, the proposed foundation of all forms of consciousness – a cross-species construct for organismic mental coherence (Panksepp, 1998b; Northoff and Panksepp, 2008). The neural instantiation of anoetic experiences of the core-self have been suggested to be related to subcortical networks in the midline of the brainstem, projecting from the periaqueductal gray to basal ganglia such as the ventral striatum, and reciprocally to medial frontal cortical regions. These brain regions, critical for consciousness, may constitute a fundamental neural substrate for the free-flow of primary-process affective states, and thereby the construction of higher-order self-representations. This primal experiences of an affective core-self, may give rise to the universal global experience of personal existence, initially unreflective (i.e., not aware of itself), but providing a foundation for higher forms of selfhood and identity through learning and increasing complexity of perceptions, cognitive appraisals, and higher-order feelings (Slaby and Stephan, 2008). We would postulate, that the anoetic self involves feelings of singularity and unity that defines a creature as a singular entity, and as a coherent human being in our species (Damasio, 2003; Prebble et al., 2013) coloring autobiographical episodic memories with affects, and determining which memories we most easily cherish and seemingly spontaneously recall and which ones we try to avoid.

Aside from the primary experience of a coherent sense of self (Damasio, 2003; Prebble et al., 2013), the higher mental representation of the self, on the other hand, reflects how memories related to critical life experiences are integrated into a higher-order affective-cognitive schema. This is supported by the ability of the brain to represent the I’ness – namely the primal viscero-somatic body – the core-self – within memory, provides for both the internally experienced and externally evident coherence of organisms (Panksepp, 1998a, 2009; Northoff and Panksepp, 2008). These primal self-structures/functions may provide a fundamental basis for learning and knowing about ourselves, since the primary-process core-of-being is densely connected with many higher association networks, like the episodic and the semantic memory system (Vandekerckhove, 2009).The higher-order semantic definition of the self or self-concept, extracted out of semantic and episodic autobiography, is the model that we typically use to explicitly organize our experiences and actions (Dennett and Westbury, 2000). However, this is granted by the intrinsic integrative circuits that already exist at brain-stem levels (Solms and Panksepp, 2012). We propose that the anoetic affective core-self-experience gives the individual a sense of self in the moment, the episodic self consisting of the unified experience of the self in the present moment together with the self as integrated with past experiences and future aspirations. It results in a diachronic self-experience, that has substantial temporal stability. It allows individuals to experience themselves as being the same person over time (Panksepp, 1998b; Panksepp and Biven, 2012). The semantic-conceptual self however, a higher-order abstraction, consists of the semantic representation of oneself over time without being permeated as richly by this affective coloring of the anoetic self.

The difference between anoetic self-experience and semantic self-concept can be illustrated by an example of an elderly woman in later stages of Alzheimer’s dementia described by Klein (2013a) who kept her anoetic self-experience intact but lost her episodic self. The women experienced a variety of memory problems typically associated with late stages of dementia (e.g., loss of personal recollections, difficulties in object naming, word finding difficulties, temporal disorientation, etc.). In contrast, interviewing revealed that she maintained a sense of herself as an entity, albeit one beset by confusion. Her anoetic self, or what Klein defines as ontological self, did not collapse as a result of her cognitive deficits and loss of access to a variety of self-relevant sources of knowledge. Rather she kept an intact subjective sense of herself as a living, experiencing entity, and behaved exactly as one would expect a conscious, subjective entity to react to the cognitive chaos engendered by the severity of the disease process that was ravaging the higher reaches of her mind (Klein, 2013b).

## Reflective Consciousness

Reflective consciousness implies primarily the capacity to have thoughts about experiences, as well as about thoughts, awareness of awareness – or meta-awareness much of which is probably unique to humans requiring expansive neocortical tissues that permit linguistic-symbolic transformation of thoughts and remembered experiences, into autobiographical perspectives (Panksepp, 2005). Mostly, explicit object-related reflective awareness springs into the foreground when the automaticity of being is not enough to handle the world. If attention within higher brain regions intensifies, reflection starts gradually to become explicit via a stream of sensorial and perceptual representations of objects in the world and events related to them. Reflective consciousness implies an explicit cognitive relationship with anoetic experiences, namely of the one who is aware and that of which one is aware. Information that becomes processed in higher awareness is then at the focus of attention, selected from competing and cooperative information channels to become more environmentally adapted and hence, cognitively predictive (Brown and Brüne, 2012). This view can also be compared with Block’s (2005) notion of “access consciousness” as it refers to the ability to manipulate information for verbal reports, planning and reasoning and crucially, for higher-order behavioral control. It thus also introduces the capacity to learn and to describe one’s own internal representations at higher cognitive levels that imply increasing “awareness” reflecting linguistic abilities of an individual ending up “knowing” something about his or her own internal states (Karmiloff-Smith, 1992; Cleeremans, 2008, 2011).

## Episodic Memory and Autonoetic Consciousness

Episodic memory is a past- and future-oriented, context-embedded neurocognitive memory system that re-presents autobiographical events from one’s past (Tulving, 2002, 2004, 2005). Tulving (1985, 1999, 2001) initially defined episodic remembering as cognitive, symbolic, and representable. It involves the capacity to reflect upon information about past events, one’s feelings during those events, and a timeline of when those experiences occurred, together with the ability to choose an event or social interaction and to recognize whether it occurred before or after some other point of reference (Robinson, 1986). Episodic memories are explicit events of one’s past, stored in a past-oriented, context-embedded, neurocognitive memory system (Tulving, 1999, 2002, 2004, 2005). Still, the consolidation and recall of these episodic memories have been immersed in and emerged from and henceforth guided by states of anoetic affective consciousness. Thereby, implicit self-relevance becomes intimately related to each individual’s unique feelings, thoughts, goals, and behaviors. Autonoetic consciousness as Klein (2013b) defines it, is intrinsic to, and dependent on episodic memory – i.e., it is constituted from “episodic” memories and sustains a relational (i.e., contingent) connection to episodic memory content.

Episodic memory constitutes autonoetic consciousness, whereas autobiographical memory involves autonoetic consciousness as long as it is episodic autobiographical memory. Autobiographical memory in itself is thus not sufficient for autonoetic consciousness. Autonoetic consciousness involves the retrieval of memories within time and context in an explicit way, constituted of self-reflective mental states. As mentioned before, it also contains the direction of attention into the future or prospection, whereas autobiographical memory can only be retrograde and semantic in its nature accompanied with noetic consciousness. Episodic memory constitutes an experience of continuity in time whereby the self is connected to previous points of time related with distinct feelings of personal agency and ownership. This highest form of consciousness makes use of the projection of the self in the context of future opportunities and possibilities, within the context of retrospective memories and prospective plans. It makes recurrent targeting of the “self” as a meta-representational mechanism where higher-order representations are targeting other possible representations of oneself (Klein et al., 2004; Cleeremans, 2008, 2011).

Tulving (2005) defined episodic memory, as differing from other forms of memory, its operations requiring an extended sense of self that can engage in mental time-travel. It targets past events, and integrates future hopes and possibilities. Reflective autonoetic consciousness, informs us about self-relevant sequences of events, enhancing the differentiation of our memories into personally relevant categories. It is accompanied with the experience – that the “I” (of agency) is the cause of “my own” thoughts and actions, or “ownership” of one’s experiences (Klein, 2013b). Awareness here depends thus also on having a high order representation of oneself as being in a particular state, experienced time and context, a process that is subtended by the prefrontal cortex (Lau and Rosenthal, 2011).

The semantic memory system, on the other hand, contains information that is remote from what most people envision as remembering, for it holds no sense of personal time or affective context (Gardiner, 2000; Markowitsch et al., 2003; Tulving, 2005; Vandekerckhove et al., 2005; Gregg et al., 2006), and does not require the explicit re-experiencing of past events (Klein et al., 2004). Noetic self-awareness is the awareness of one’s self in semantic ways, characterized by personality traits and factual self-knowledge, whereas autonoetic self-consciousness refers to explicit self-awareness in a specific affectively tinged time-space context and continuum.

## The Anoetic Warmth of Remembrance

The episodic remembrance of one’s own life experiences is filled with the pervasive affective consciousness associated with specific times, places, and events. It involves a detailed sensory affective-perceptual re-experiencing of events, and this is importantly related to anoetic consciousness. The reliving of the subjective experiences is closely linked to an affective evaluation of the significance of these past experiences for oneself and with respect to one’s previous and present position in the world (Markowitsch and Staniloiu, 2012). In James’s (1890) view, episodic remembering and autonoetic awareness is described as “remembrance” with “warmth and intimacy” referring, we propose, to the phenomenal, self-referential flavor of anoetic consciousness and to the remembering of past value-laden episodic events or autonoetic awareness. One can actually feel oneself as if one was in previous scenarios. Episodic memory is especially dependent on the encoding and consolidation of many differentiated cognitive aspects each with many elaborations, with the remembered anoetic, affective, and environmental contexts in which they occurred. This memory system is enriched by characteristics such as the feeling of selfhood and agency, vividness; temporality; contextuality of color, taste, and smell; affective richness; and cognitive subtlety. The richness of affective coloring and value presumably arises from a host of affective systems, coded by diverse neuropeptides, which gives valuative richness to life (Panksepp, 1998a; Solms and Panksepp, 2012). As Klein (2013b) discusses, referring to Zahavi (2005) and James (1890), episodic memory connects to the past not by logical inference like it is the case in semantic memory, but by “pre-reflective directly givenness” (e.g., Zahavi, 2005). It is a feeling that “my current mental state stands for and thus is representative of an experience in my personal past” (e.g., James, 1890). Accompanied by anoetic consciousness it reflects a sense of affective attachment to my past, reflected in this warmth and intimacy or feeling of familiarity which is part of the valuative subjective quality of the mental event. And this feeling accompanying episodic recollection is made possible by episodic memory’s association with autonoetic awareness. It follows that impairment in any of these episodic crucial characteristics or components, such as self-reflection, self-agency, self-ownership, and personal temporality should produce, in varying degrees, specific circumscribed impairments in episodic recollection (Klein, 2013b). Klein (2013b) illustrates this by R.B. who suffered from a serious head injury from a car accident, a number of cognitive impairments such as retrograde and anterograde amnesia for events in close temporal proximity to the accident. R.B. was still able to remember particular incidents from his life accompanied by clear temporal, spatial, and self-referential content. However, he no longer felt ownership of the memories, warmth and intimacy, or autonoetic consciousness.

In contrast, semantic memories do not have these abundant dependencies on anoetic consciousness – neither the contextual variables nor associated self-referential dynamics that give episodic memories such personal closeness and feelings of ownership and selfhood. Semantic memory can be self-referential and spatial (Klein, 2013a) as it consists of factual knowledge about the self and world abstracted from the specific episodic contexts from which the knowledge had been acquired. This distinction between episodic and semantic memorial experiences can also be seen, as Klein (2013b) described, as one of differences in manner of acquaintance (e.g., Russell, 1916/1999). We are acquainted with semantic pastness indirectly via inference, whereas our acquaintance with episodic pastness is directly given as the phenomenological feeling that we are re-living our past (Klein, 2013b).

Severe impairment of episodic memory retrieval, particularly of the emotional information from specific autobiographical content has been found in patients with lesions that include amygdaloid complexes and related circuitry with forebrain regions (Markowitsch and Staniloiu, 2012). The amygdala’s main function is to affectively charge cues, especially in aggressive, fearful, and sexual domains, so that explicit or implicit memory events of a specific anoetic significance can be successfully searched within the appropriate neural nets and thereby re-activated (Markowitsch and Staniloiu, 2012). Emotionally stressful life events impact the amygdala and the hippocampus – areas with a rich density of glucocorticoid receptors – so as to consolidate especially stressful memories. The Papez circuit and the basolateral limbic loop (mediodorsal nucleus of the thalamus, subcallosal area, amygdala, and interconnecting fiber) are also involved. The Papez and the basolateral limbic circuit represent a group of “bottleneck-structures” that are interconnected and of high relevance for the extraction of the affective, somatosensorial, and social significance of new incoming information (Markowitsch and Staniloiu, 2012). In contrast, when damage involves just the semantic memory system, especially of the hippocampus, patients may forget personal facts and beliefs – where and when they were born, details of their physical appearance, but their anoetic self remains relatively intact.

## Conclusion and Summation: Unknowing Anoetic Consciousness Guides the Emergence of Knowing Consciousness

Anoetic consciousness has here been envisioned as a stream of pre-reflective affective and sensorial perceptual consciousness essential for the waking state of the organism in the absence of an explicit self-referential awareness of associated cognitive contents. This state of affective consciousness acts as a bridge that may take us from deeply unconscious information processing and primary-process affective and perceptual consciousness toward the possibility of knowing (noetic) levels of consciousness situated within the basal ganglia that mediate learning and memory and higher regions of the brain, such as the neocortex which allows for mental time-travel from the permutations of these memories. In other words, unknowing consciousness, namely anoetic consciousness, allows various primordial affective feelings, and the related affective information processing of learning and memory mechanisms, especially in brain structures known as the basal ganglia (amygdala, nucleus accumbens, bed nuclei of the stria terminalis) to connect up with knowing noetic, and especially, autonoetic consciousness. Anoetic self-experience influences global phenomenal experiences – unconditional states of being – which not only modulate the affective tone of one’s behavior, but provide critical information for learning and memory. As primarily pre-conceptual phenomena, anoetic affective experiences, can be differentiated into various intrinsic valuative processes of the brain (emotional, homeostatic, and sensory affects) that become continuously part of actual information processing and existential perception of the world. Later in development, when reflection is possible, those primal affects act as free-flowing streams underlying the more cognitively detailed aspects of our continuously ongoing cognitive-noetic and autonoetic information processing as thoughts, images, fantasies, expectations, and anticipations. This is very similar to the “free flow of consciousness” that James (1890) talked about. In contrast to noetic consciousness, autonoetic consciousness refers to the reflective capacity to mentally represent a continuing existence one that is embedded in specific episodic contexts and associated with remembered experiences with affective quality – from “warmth and intimacy” to “dread and alienation.”

Noetic consciousness is associated with the knowledge that specific facts have happened in the past, but it has no access to a fully resolved, affectively rich awareness of one’s ongoing subjective experience. Noetic self-awareness is the awareness of one’s self in semantic ways, characterized by personality traits and factual self-knowledge, whereas autonoetic consciousness refers to explicit self-awareness, and/or the explicit awareness of something or someone else in a specific affectively tinged time-space context. This highest episodic self-referential form of consciousness makes use of the projection of the self in the context of future opportunities and possibilities, within the context of retrospective memories and prospective plans. As a self-generative, self-knowing state, attention in autonoetic consciousness can thus be directed to memories of the past – of being retrospective, with past anoetic flows of consciousness available in the “real-time” present, and planning and dreaming of the future in a prospective form of autonoetic consciousness. It is accompanied with a sense of personal agency; that is, the belief that I am the cause of my thoughts and actions, a sense of personal ownership; that is, the feeling that my thoughts and acts belong to me, and the ability to think about time as an unfolding of personal happenings centered about the self (Klein, 2013a).

This form of mentalizing, surely most highly developed in humans, is heavily mediated by medial temporal lobe (hippocampal) and frontal lobe evolution and microstructure (Buckner and Carroll, 2007; Fleming et al., 2010). Neural correlates of noetic (knowing) consciousness relate to various memory abilities, especially declarative (factual) memory, whereas anoetic consciousness is heavily linked, to raw sensorial and perceptual abilities, to various subcortical affective processes, and intrinsic affective value structures, and hence, is more related to limbic and paralimbic structures associated intrinsically with the more implicit free-flow of affective consciousness. We suggest that what came first in the evolution of consciousness, namely the anoetic forms, sustain primacy in the construction of the higher, noetic and especially autonoetic forms that are of such critical importance for our individual decision-making abilities and autobiographical existences.

## Conflict of Interest Statement

The authors declare that the research was conducted in the absence of any commercial or financial relationships that could be construed as a potential conflict of interest.

## Footnotes

^1^There are some resemblances here to Ned Blocks, “phenomenal,” and “access” consciousness, which has been discussed neuroscientifically with respect to the neural correlates of consciousness (Block, 2005). However, his view does not conceptualize affective states and their role in learning, nor provide a way to conceptualize how higher forms of consciousness emerge from the lower anoetic and noetic levels that we think are essential for conceptualizing the hierarchical evolutionary strata of the brain-mind. Anoetic consciousness can be differentiated from Block’s (1998, 2005) definition of “phenomenal consciousness,” namely what it is like to be in a first order phenomenal state, encompassing all first-person conscious experiences, seemingly at both anoetic and noetic levels of experiencing, but need to be overtly reportable (hence a human phenomenon), while for us anoetic consciousness, by its very nature, is considered to reflect rudimentary implicit affective and perceptual brain states that are valenced, and can be possible studied in animal models because one can evaluate the affective properties of brain states as with DBS procedures. Related feelings are never unexperienced in humans, and DBS research provides experimental access to such shared feeling processes.
